# Hippo Pathway in Cancer, towards the Realization of Hippo-Targeted Therapy

**DOI:** 10.3390/cancers10100383

**Published:** 2018-10-12

**Authors:** Yutaka Hata

**Affiliations:** Department of Medical Biochemistry, Graduate School of Medical and Dental Sciences, Tokyo Medical and Dental University, Tokyo 113-8519, Japan; yuhammch@tmd.ac.jp

Transcriptional co-activators, YAP/TAZ, have attracted attention as promising targets in cancer therapy. YAP/TAZ are negatively regulated by the tumor suppressor Hippo pathway. In human cancers, YAP/TAZ are frequently hyperactivated as a consequence of dysregulation of the Hippo pathway. Mounting clinical evidence demonstrates a correlation between the activation of YAP/TAZ and poor prognosis. YAP/TAZ co-operate with transcription factors, TEADs, to induce epithelial-mesenchymal transition (EMT), and to cause resistance to drug treatment. It is reasoned that the inhibition of YAP/TAZ should improve prognosis in cancer patients. Researchers devote a great deal of effort to developing inhibitors of YAP/TAZ, and face many exciting opportunities as well as challenges. The aim of this special issue is to offer the basis for discussion about how to put the Hippo-targeted cancer therapy into practice. This paper is not intended to provide a comprehensive overview of the research on the Hippo pathway. All contributors were requested to write their manuscripts freely. Therefore, there is divergence in the styles of the presented papers. Some authors focus on their own research and introduce original findings as commentaries. Nevertheless, with twelve papers and reader-friendly introductions, the issue covers a significant part of the current achievements in this field.

TEADs are the most important partners of YAP/TAZ. The interaction between YAP/TAZ and TEADs is regarded as a good candidate for cancer therapeutic intervention. Holden and Cunningham discuss how YAP/TAZ-TEAD interface is targeted [1]. Notably, although it is the prevailing view that transcription factors are unsuitable as drug targets, the identification of the lipid pocket in TEAD has drastically changed this view. Gibault et al. also introduce their own efforts toward the discovery of inhibitors of YAP-TEAD interaction by using virtual screening [2].

In the classical model, YAP/TAZ are regulated by the kinase cassette, MST-Sav1-MOB1-LATS. However, we now know that many other pathways regulate YAP/TAZ. Some of them work via the kinase cassette, while others directly modulate YAP/TAZ activity. Thus, YAP/TAZ can be inhibited in various ways. Warren et al. efficiently and conceptually review the possible methods [3]. Malignant mesothelioma (MM) is a representative cancer with dysregulation of the Hippo pathway. Sekido reviews how the Hippo pathway is disturbed in MM, and then discusses methods to suppress YAP/TAZ [4]. Reading these two reviews, we can obtain an overview of upstream strategies. Hardness is one of notable characters of cancer. YAP/TAZ respond to stiffness of extracellular matrixes (ECMs). Prevention of ECM stiffening is a rational choice to inhibit YAP/TAZ. Chakraborty and Hong highlight agrin, which enhances ECM stiffness, as a novel up-stream regulator of YAP in liver cancer; they discuss the potential therapeutic value of targeting agrin [5]. Rivas et al. propose WIP as a regulator of YAP/TAZ and discuss the role of WIP-YAP/TAZ in glioma with p53 mutations [6]. The expression levels of components of the Hippo pathway are regulated by E3 ligases. As one E3 ligase modulates more than one protein, it is not easy to predict the outcome of the targeting of E3 ligases. However, reading the review by Nguyen and Kugler, we understand that certain E3 ligases are promising targets in cancer therapy [7]. They also briefly comment on other modifications of lysine residues, which are potential targets to suppress YAP/TAZ activity.

Targeting YAP/TAZ-TEAD is considered safe, because YAP/TAZ and TEADs are not necessary for tissue homeostasis in adults. Even so, to minimize unexpected side effects, downstream strategies which target genes up-regulated by YAP/TAZ-TEAD specifically in cancer cells are desirable; Warren et al. and Sekido again discuss these strategies [3,4]. This approach is important in the treatment of epithelioid hemangioendothelioma (EHE), which is reviewed by Lamar et al. [8]. In 90% of EHE, TAZ is fused to calmodulin binding transcription activator 1 (CAMTA1), and drives endothelial cell proliferation. As TAZ-CAMTA1 is not regulated in the same manner as TAZ, upstream strategies are useless for EHE. In contrast, as the oncogenic property of TAZ-CAMTA1 depends on TEAD, and transcriptional changes induced by TAZ-CAMTA1 and TAZ are overlapped, the direct targeting of TAZ-TEAD and downstream strategies are expected to be useful for EHE. In other words, EHE, although it is rare and may be an outlier, can be a leading model for evaluating the efficiency of newly-developed therapies before they are applied to more common cancers with hyperactive TAZ.

We need to consider how to evaluate the effect of the Hippo-targeted therapy. Warren et al. expound the roles of YAP/TAZ in cancer metastasis [3]. We may find the most remarkable effect of YAP/TAZ inhibition as decreased incidences of metastasis. Lo Sardo et al. extensively and minutely review the dysregulation of the Hippo pathway and oncogenic roles of activated YAP/TAZ in lung cancer [9]. This review also includes their own striking discovery of synergism between YAP and mutated p53. They note that driver genes for lung cancer such as KRAS and EGFR regulate YAP/TAZ, and that YAP increases drug resistance in cancer. To correctly assess effect of YAP/TAZ inhibitors, we may need to use them with other anti-cancer drugs. YAP/TAZ inhibitors themselves may not lead to a decrement in tumor size. One of current topics in cancer therapy is immunotherapy such as PD-1/PD-L1 blockade. Taha et al. discuss the involvement of the Hippo pathway in immunity [10]. YAP induces cytokine expression and recruits immune-suppressive cells. YAP/TAZ upregulate PD-L1 expression. In this way, YAP/TAZ make cancer cells evade immune surveillance. That is, YAP/TAZ inhibitors may exhibit significant effect on tumor progression after the immune response properly works in patients. Even if statins suppress YAP/TAZ, who could expect that tumors diminish in response to statins? We should not stick to the dream that YAP/TAZ inhibitors instantly abolish tumors. We need to be patient in our evaluations of YAP/TAZ inhibitors.

We cannot neglect a couple of paradoxical twists in the implication of the Hippo pathway and YAP/TAZ in the pathophysiology of cancer. In spite of the above-mentioned roles of YAP/TAZ in cancer immunity, LATS-depleted mouse cancer cells secrete vesicles including nucleic acids, enhance immune response in vivo, and fail to form tumors in immunocompetent mice. We must be careful to extrapolate in vitro findings to human patients. Another twist is the tumor suppressive role of YAP. In this issue, Ishihara and Nishina present their study to show that cells harboring constitutively active YAP are excluded from the cell layer when surrounded by normal cells, and assign a tumor suppressor role to YAP [11]. Retrospectively, we found that YAP was initially studied as a partner with pro-apoptotic p73; actually, YAP causes apoptosis in certain cells. Therefore, we must selectively apply YAP/TAZ inhibitors to appropriate cancers.

Finally, Elisi et al. discuss the repurposing of already-approved drugs to the modulation of YAP/TAZ and TEAD [12]. This approach is probably the fastest and the most realistic way to implement the Hippo-targeted therapy. The effect of repurposed drugs may not be remarkable as specifically developed molecular-targeted drugs, but those already-used drugs can be safely used and immediately tested. Applying repurposed drugs to well-selected cancers, whose malignant properties clearly depend on YAP/TAZ, such as MM and EHE, will provide a good starting point for understanding how to use and evaluate YAP/TAZ inhibitors. We need to deliberately design strategies towards the realization of the Hippo-targeted cancer therapy.

The planning of this special issue was sparked by two meetings held in 2017: the Telluride Science and Research Center workshop “YAP/TAZ and TEAD: At the crossroads of cancer” organized by Guy Weinberg, Peter Salamon, and John Lamar, and the EMBO Workshop “The Hippo pathway across species and disciplines” organized by Nic Tapon, Giovanni Blandino, and Stefano Piccolo. I would like to express my deepest gratitude to the organizers and participants who kindly responded to my request and contributed their precious manuscripts to this issue.
